# A Structural Equation Modeling of Mental Health Literacy in Healthcare Students

**DOI:** 10.3390/ijerph182413264

**Published:** 2021-12-16

**Authors:** Chia-Min Lu, Yin-Ju Lien, Hsing-Jung Chao, Hui-Shin Lin, I-Chuan Tsai

**Affiliations:** Department of Health Promotion and Health Education, National Taiwan Normal University, 162, Heping East Road Section 1, Taipei 106, Taiwan; chiaminluu@gmail.com (C.-M.L.); chaoshinlon1@gmail.com (H.-J.C.); huishin00725@gmail.com (H.-S.L.); franktsai0425@gmail.com (I.-C.T.)

**Keywords:** mental health literacy, healthcare students, help-seeking, parallel mediation, theory-based construct

## Abstract

**Background:** There is a high prevalence of mental illness among healthcare students, and most students with mental health problems are reluctant to seek help from mental health professionals. Help-seeking is a component of mental health literacy (MHL). Although MHL is conceptualized as multi-dimensional, a theory-based multi-construct of MHL is still lacking. We aimed to build a theory-based multi-construct of MHL to explore the pathways of help-seeking. **Methods:** The data were obtained from a survey on MHL among healthcare students in 2018 (*n* = 1294). The Mental Health Literacy Scale for Healthcare Students was used to measure the maintenance of positive mental health, recognition of mental illness, mental illness stigma attitudes, help-seeking efficacy, and help-seeking attitudes. Descriptive analysis and structural equation modeling (SEM) were conducted. **Results:** The findings of the SEM model indicated recognition of mental illness had a positive direct effect on both help-seeking efficacy and maintenance of positive mental health. Additionally, help-seeking efficacy fully mediated the relationship between recognition of mental illness and help-seeking attitudes. **Conclusions:** Help-seeking efficacy plays a significant role in healthcare students’ willingness to seek professional help when mental health care is needed. Accordingly, improving help-seeking efficacy strategies would increase the use of mental health services and contribute to the prevention of mental health problems.

## 1. Introduction

College students often find themselves suffering from mental health problems [1], with clinical phenotypes produced by stressful life events [2]. Individuals in this period are usually responsible for their own decisions for the first time; therefore, they are prone to mental health problems [3]. In particular, healthcare students apply much heavier academic pressure caused by medical subjects and training than students majoring in other professions [4], with a relatively high prevalence of mental disorders (33.9% to 58.7%) [5,6,7]. Unwillingness to seek treatment for mental health problems has negative impacts (e.g., interpersonal difficulties and dropping out of school) [8], affecting future clinical practice and healthcare quality [9]. However, only 11.0% to 15.7% of healthcare students with mental illness seek professional help [10,11].

Mental health literacy (MHL) plays an important role in seeking mental health professional help [12]. Based on Jorm’s definition [13], Kutcher and Wei [14] extended the previous definitions of MHL to include four components: (1) maintenance of positive mental health; (2) recognition of mental disorders; (3) mental illness stigma attitudes; and (4) help-seeking efficacy (i.e., knowing when and where to receive mental health services). Furthermore, help-seeking attitude was added as a component of MHL [15] because it was a powerful predictor of help-seeking behavior [16]. The abovementioned components of MHL are related to attitudes toward seeking professional help [17,18,19]. Exploring the relationship among factors influencing help-seeking attitudes is helpful in improving attitudes toward seeking help [20] and enhancing help-seeking behavior [12]. The relationship among the factors affecting help-seeking attitudes should be based on theories because theories can articulate how they interact to predict a specific outcome [21]. In addition, it has been proposed that MHL is a multi-construct theory rather than a multi-dimensional construct [22].

Several theories and models have been applied to mental health help-seeking. First, the model for mental health help-seeking shows three stages of the pathway for seeking help from mental health professionals: problem identification, decision to seek help, and service selection [23]. It is worth noting that the decision to seek help is influenced by mental illness stigma. In the second approach, help-seeking theory conceptualizes help-seeking as a multi-step process, which begins with the individual’s awareness of mental health problems, followed by the identification of appropriate help and available sources to access, and ending with the willingness to seek help [24]. In particular, the identification of appropriate mental health sources means that individuals know where and when to receive mental health services, a concept similar to help-seeking efficacy. The final theory is the health belief model, which posits that the decision to perform a behavior is dependent on the individual’s perceived threat of illness and its severity, in addition to the perceived barriers and benefits of the behavior [25]. Based on the above theories, the process of help-seeking generally starts with recognition of mental health disorders and finishes with a help-seeking attitude. Most studies indicated that recognition of mental illness was positively associated with help-seeking attitudes [20,26,27], whereas a study showed that individuals with better knowledge of mental health symptoms were unwilling to seek professional help [28]. Accordingly, we infer that the potential mediators between the relationship of recognition of mental illness and help-seeking attitudes may account for the inconsistent findings.

Some factors are related to recognition of mental illness and help-seeking attitudes, such as mental illness stigma attitudes, help-seeking efficacy, and the maintenance of positive mental health. First, it has been indicated that individuals with better knowledge of mental health disorders have less stigma toward mental illness [20,29,30]. Stigma is also negatively related to help-seeking attitudes [31], showing that individuals with higher stigma are unwilling to seek professional help [32]. Second, both the general public and healthcare professionals with a better ability to identify mental disorders learn more information about mental health care resources [33,34]. Moreover, individuals with higher knowledge about mental health services have a positive attitude toward seeking help from mental health professionals [10,35]. In addition to mental illness stigma attitudes and help-seeking efficacy, the maintenance of positive mental health plays an important role in the process of seeking help from mental health professionals [36]. It has been shown that recognition of mental illness has a positive correlation with the maintenance of positive mental health, which indicates that enhancing the ability to recognize mental illness improves the information about mental health care resources [37,38]. Furthermore, individuals with more knowledge about the maintenance of positive mental health are willing to seek help for mental health problems [39].

**Figure 1:**
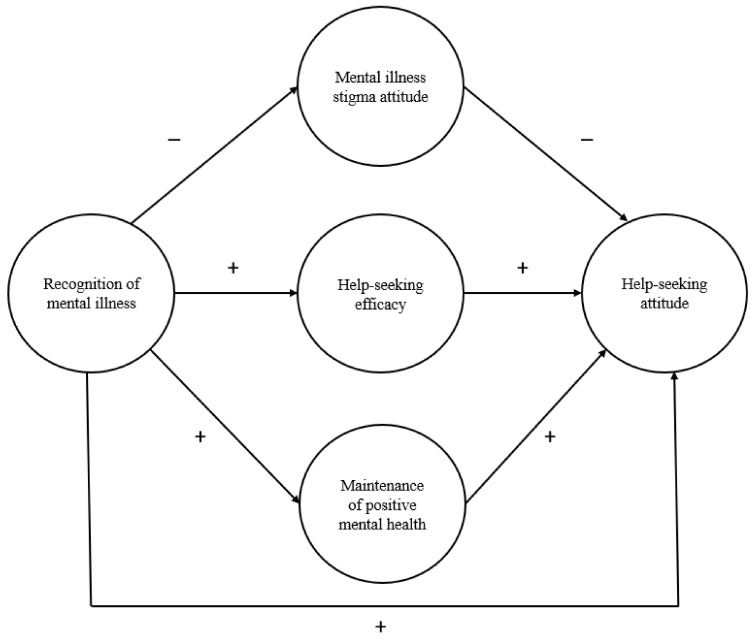
Hypothesized mediation model of five components of mental health literacy.

To understand the process of mental health help-seeking among healthcare students, we aimed to propose, build, and validate a theory-based construct of MHL. Based on previous studies, a hypothesized model linking recognition of mental illness to help-seeking attitudes was proposed (Figure 1): higher recognition of mental illness is directly related to better help-seeking attitudes, less stigma, greater help-seeking efficacy, and more maintenance of positive mental health (Hypothesis 1); less stigma, higher help-seeking efficacy, and more maintenance of positive mental health are directly related to better help-seeking attitudes (Hypothesis 2); recognition of mental illness has an indirect effect on help-seeking attitudes through mental illness stigma attitudes (Hypothesis 3); recognition of mental illness has an indirect effect on help-seeking attitudes through help-seeking efficacy (Hypothesis 4); and recognition of mental illness has an indirect effect on help-seeking attitudes through the maintenance of positive mental health (Hypothesis 5).

## 2. Methods

### 2.1. Participants

We used data from the MHL survey in Taiwan [15]. The participants were older than 20 years in 11 universities with departments of medical and public health in Taiwan. The study was conducted between April and June 2018 and 1685 individuals were invited to participate in this study. A total of 354 respondents refused to participate and 37 were excluded after a data quality check because of their repeated endorsement of the same response option throughout the scale. Finally, the sample consisted of 1294 healthcare students, with 970 medical students (74.96%) and 324 public health students (25.04%). The response rate is 76.80%. More than half of students were men (55.8%); 1060 individuals were junior and senior undergraduate students (81.9%), with a mean age of 22.79 years. More than half of the participants had no specific religion (51.5%), and their monthly household income was between TWD 100,000 and 200,000 (27.1%). Ethical approval for this study was obtained from the National Taiwan Normal University Institutional Review Board (ID:NTNUREC-2017HS006).

### 2.2. Measures

We used the Mental Health Literacy Scale for Healthcare Students (MHLS-HS) [15], which comprised 26 items in the paper version. It has demonstrated good internal consistency (Cronbach’s α = 0.81) and convergent and discriminant validity [15]. The measurement was divided into five subscales: maintenance of positive mental health, recognition of mental illness, mental illness stigma attitudes, help-seeking efficacy, and help-seeking attitudes.

The maintenance of positive mental health subscale comprised 10 items to assess knowledge about the maintenance of positive mental health. All items used a 5-point Likert rating scheme, ranging from 1 (strongly disagree) to 5 (strongly agree). Higher scores indicated better levels of positive mental health information. Internal consistency was good (Cronbach’s α = 0.87) [15].

The subscale of mental illness recognition included items that identify serious and common mental disorders (schizophrenia, anxiety, depression, and substance-related and addictive disorders). The participants were asked to indicate the extent to which they agreed with each statement describing the symptoms and characteristics of someone with a specific mental illness. Responses were rated on a 5-point Likert scale, ranging from 1 (strongly disagree) to 5 (strongly agree). Higher scores indicated a better ability to correctly recognize the signs and symptoms of mental disorders. It demonstrated acceptable internal consistency (Cronbach’s α = 0.70) [15].

The subscale of mental illness stigma attitudes measured the concepts of stigma, including public stigma (i.e., belief that a person is socially unacceptable by others), dangerousness (i.e., patients with mental disorders are dangerous), and emotional reactions (i.e., fear of individuals with mental illness). There were six items rated on a 5-point Likert scale, ranging from 1 (strongly disagree) to 5 (strongly agree), with higher scores indicating higher levels of mental illness stigma attitudes. The internal consistency reliability was 0.76 [15].

The help-seeking efficacy subscale included items regarding knowing who, where, and when to seek mental health professional help. There were three items rated on a 5-point Likert scale, ranging from 1 (strongly disagree) to 5 (strongly agree), with higher scores indicating greater knowledge of mental health resources. The internal consistency reliability of the subscale was good (Cronbach’s α = 0.81) [15].

The subscale of help-seeking attitudes measuring the attitude toward seeking professional help from psychological difficulties was composed of three items on a 5-point Likert scale. Responses to the subscale ranged from 1 (strongly disagree) to 5 (strongly agree). Higher scores indicated a more positive attitude toward seeking mental health professional help. The subscale demonstrated acceptable internal consistency (Cronbach’s α = 0.72) [15].

### 2.3. Data Analyses

SPSS version 23 was used for the descriptive and other statistical analyses. AMOS version 21 [40] was used for the confirmatory factor analysis and structural equation modeling (SEM). Descriptive analyses were conducted using the means and standard deviations (SD) of the observed variables. Skewness and kurtosis were calculated to assess data normality. To satisfy the assumption of univariate normality, the absolute values of the skewness and kurtosis of the observed variables should be less than 2 and 7 [41], respectively. Multicollinearity was examined by tolerance and variance inflation factor (VIF).

Before performing SEM, the multivariate normality should be tested. It was established using Mardia’s coefficient using Bollen’s method [42]. Accordingly, when Mardia’s coefficient is less than *p* (*p* + 2) (where *p* is the number of observed variables), it shows multivariate normality. SEM with a maximum likelihood estimation was conducted to test the fitness of the hypothesized model for the maintenance of positive mental health, recognition of mental illness, mental illness stigma attitudes, help-seeking efficacy, and help-seeking attitudes. To assess the goodness of fit of the proposed model, the following indices were used [41,43,44]: the likelihood ratio (χ^2^/df), goodness-of-fit index (GFI), comparative fit index (CFI), square error of approximation (RMSEA), and standardized root mean squared residual (SRMR). The procedure of the bootstrap bias correction procedure was used to examine the mediation effects [45]. Standardized coefficients and 99% confidence intervals were based on 5000 bootstrap resamples. Because the number of samples in this study was more than 1000, we set the significance level of each statistical test as *p* < 0.01 to reduce type II errors [46,47]. The statistical significance of the direct, indirect, and total effects is expressed as a z-score higher than 2.58 and a *p*-value less than 0.01.

## 3. Results

### 3.1. Descriptive Analysis

There was no significant difference in the scores of the five subscales of the MHLS-HS between medical students (*n* = 970) and public health students (*n* = 324). Accordingly, we used the MHLS-HS scores of all students, including medical and public health students, for subsequent analyses. The values of the five components of MHL met the criteria for skewness (range between −0.67 and 0.24) and kurtosis (range between −0.14 and 0.89), indicating that the data satisfied the assumption of normal distribution. Tolerance was 0.92–0.95 (>0.10), and VIF was 1.04–1.09, which were smaller than the criterion value of 5 [43], indicating no evidence of multicollinearity among these variables (Table 1).

### 3.2. Measurement Model

**Figure 2:**
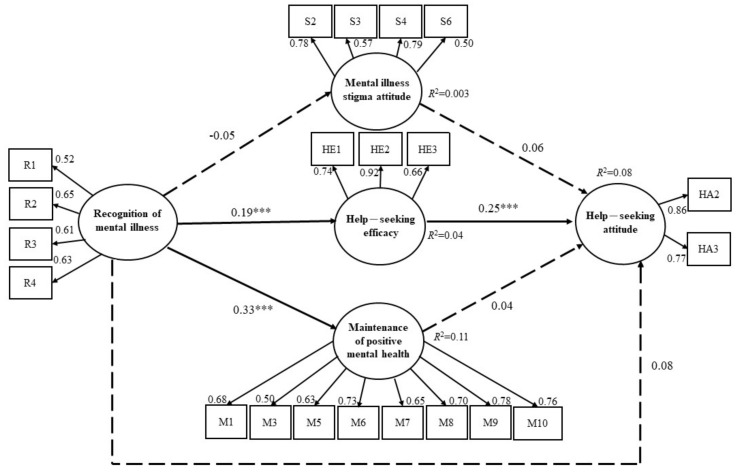
Path diagram of the mental health literacy structural equitation model in healthcare students. Note: Solid lines indicate significant paths; dashed lines show paths did not reach the level of statistical significance. *** *p* < 0.001.

**Figure A1:**
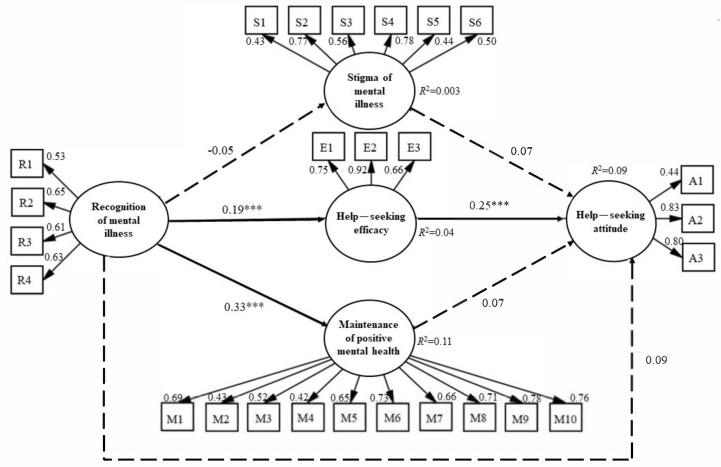
Path diagram of the mental health literacy structural equation model of 26 items in healthcare students. Note: Solid lines indicate significant paths; dashed lines show paths did not reach the level of statistical significance. *** *p* < 0.001.

Although the MHLS-HS, including 26 items, showed good content validity, internal consistency, and construct validity [15], there were five items with factor loadings less than 0.5. The aim of the present study was to verify the theory-based multi-construct of MHL, and more rigorous criteria for construct validity were used. Generally, researchers consider that items with factor loadings <0.50 are removed first [48,49,50]. Hence, we reserved only 21 items of the MHLS-HS, with factor loadings over 0.5, for SEM analysis. In this study, Cronbach’s alpha coefficients for the five subscales (21 items) ranged from 0.70 to 0.87, with an overall α-value of 0.74. Figure 2 shows that the standardized factor loadings of all indicators in the measurement model were 0.50–0.92, which satisfied the criteria. The fit indices were χ^2^/df = 4.13, RMSEA = 0.04, SRMR = 0.03, GFI = 0.94, and CFI = 0.93, suggesting that the data fit the measurement model well. Similar findings were found when using the original 26 items of the MHLS-HS (see Appendix A Figure A1).

### 3.3. Structural Model

Mardia’s coefficient was 115.71; given that *p* = 21, this was less than the value for *p* (*p* + 2) = 483, confirming multivariate normality. The model fit indices indicated that this model fit the data (χ^2^/df = 4.33, RMSEA = 0.04, SRMR = 0.04, GFI = 0.94, and CFI = 0.93). Figure 2 shows that recognition of mental illness had a direct effect on help-seeking efficacy (β = 0.19, *p* < 0.001) and maintenance of positive mental health (β = 0.33, *p* < 0.001), showing that individuals had greater ability to recognize mental illness, and greater knowledge about mental illness care resources, and positive mental health. Furthermore, help-seeking efficacy was found to directly influence help-seeking attitudes (β = 0.25, *p* < 0.001), revealing a higher help-seeking efficacy and a more positive attitude toward seeking mental health professional help. Comparable results were observed when using the original 26 items of the MHLS-HS (see Appendix A Figure A1).

### 3.4. Mediating Effect Testing

To assess whether the mediating effects were statistically significant, we followed the criteria showing that the indirect effect was significant, and the confidence interval did not include 0. The mediating effects of three variables (mental illness stigma attitudes, help-seeking efficacy, and maintenance of positive mental health) between recognition of mental illness and help-seeking attitudes were examined to determine statistical significance using the bootstrap assessment procedure. The results are shown in Table 2, showing that the indirect effect of help-seeking efficacy was significant (β = 0.04, *p* < 0.001; CI = 0.02 to 0.08). However, the indirect effects of mental illness stigma attitudes (β = −0.003, *p* = 0.14; CI = −0.02 to 0.003) and the maintenance of positive mental health (β = 0.01, *p* = 0.20; CI = −0.02 to 0.05) were not significant. In summary, only help-seeking efficacy mediated the relationship between recognition of mental illness and help-seeking attitudes, whereas the direct path from recognition of mental illness to help-seeking attitudes was not significant (β = 0.08, *p* = 0.07; CI = −0.03 to 0.18). This indicated a full mediating effect of help-seeking efficacy on the relationship between recognition of mental illness and help-seeking attitudes. The similar findings were reported when we conducted mediating effect testing with the original 26-item scale (see Appendix Table A1).

## 4. Discussion

This is the first study to build a theory-based multi-construct of MHL to examine the relationship among the five components of MHL, providing a more thorough understanding of the relationship between the maintenance of positive mental health, recognition of mental illness, mental illness stigma attitudes, help-seeking efficacy, and help-seeking attitudes within healthcare students. We found that recognition of mental illness was positively correlated with help-seeking efficacy and the maintenance of positive mental health. Additionally, healthcare students with better knowledge of mental health resources were willing to seek help for mental health problems. Finally, improving recognition of mental illness led to a greater help-seeking efficacy, which eventually enhanced attitudes toward help-seeking.

Several pathways were found to have direct effects in the present study. First, individuals with better recognition of mental disorders enhanced their help-seeking efficacy, which is consistent with previous studies [51,52]. Individuals with knowledge regarding symptoms and treatment of mental illness consider that psychiatric disorders are the same as physical illness [53], which leads to the belief that patients with mental illness should receive medical treatment. Second, we found that recognition of mental disorders directly influenced the maintenance of positive mental health, which is in line with previous literature [37,54]. Previous studies revealed that healthcare students with interest in the subject matter tend to search for relevant information [55]. Accordingly, healthcare students acquire knowledge of mental health in professional courses, which may result in a better understanding of the maintenance of positive mental health. In the third pathway, we found that individuals with better information regarding mental health services held more positive help-seeking attitudes, which is consistent with those reported earlier [56,57]. It is worth noting that previous studies have applied the theory of planned behavior (TPB) [58] to explain the process of help-seeking for mental health problems, with the results indicating that help-seeking efficacy predicted help-seeking intention [59,60,61]. Perceived behavioral control is an element of TPB, which is defined as an individual’s perception of the skills, resources, and opportunities needed to perform a behavior [58]. Better perceived behavioral control had a positive impact on behavioral intention [62]. Thus, individuals with greater information about mental health services may promote help-seeking attitudes [60].

In the parallel mediation model of MHL, three indirect effects were examined. First, help-seeking efficacy was found to have a fully mediating role in the relationship between recognition of mental illness and help-seeking attitudes. This result is consistent with help-seeking theory [24], implying that understanding mental health resources is a potential mediator between awareness of mental illness symptoms and willingness to seek help. Moreover, help-seeking efficacy has been considered a facilitator in the help-seeking process [63]. Individuals with better knowledge of mental illness resources can effectively obtain mental health services [13].

Second, the results indicated that mental illness stigma attitudes did not mediate the relationship between recognition of mental illness and help-seeking attitudes. Only a handful of studies have examined the mediating role of mental illness stigma attitudes on the association between awareness of mental health problems and willingness to seek help, with mixed findings to date [12,20]. A possible reason that mental illness stigma attitude did not serve as a mediator is that recognition of mental illness was not related to mental illness stigma attitude in this study. Recognition of mental illness is recognized as a knowledge dimension, whereas mental illness stigma attitude is considered an attitude dimension. Studies show that attitudes are often emotional and implicit rather than logical [64]. The change in recognition of mental illness without a concurrent change in mental illness stigma attitudes illustrates a potential disconnection between thoughts and feelings [65]. In addition, the results of this study indicated that mental illness stigma attitude was not associated with help-seeking attitudes. Of note, the impact of the different types of stigma on help-seeking attitudes is distinct [20]. There are two main types of stigma associated with mental health problems: personal stigma and self-stigma [66]. Personal stigma refers to how people view and treat others with mental illness [67], whereas self-stigma refers to negative attitudes toward people with mental illness with regard to their own condition (e.g., internalized shame) [68]. In the current study, the concept of mental illness stigma attitude was considered as personal stigma. A systematic review showed that help-seeking attitudes were related to self-stigma rather than personal stigma [31]. Seeking help is for one’s own mental illness, and the way a person interacts with other people with mental illness may not have a significant relationship with one’s attitude toward seeking mental health help [20].

Finally, we found that the maintenance of positive mental health did not mediate the relationship between recognition of mental illness and help-seeking attitudes. Although recognition of mental illness had a direct effect on the maintenance of positive mental health, the maintenance of positive mental health was not found to correlate with help-seeking attitudes. The knowledge, attitude, and practice (KAP) model posits that knowledge positively influences an individual’s attitude; and attitude, in turn, influences practice or behavior [69]. Moreover, previous studies used the KAP model to explore the process of help-seeking among patients with diabetes. The results indicated that patients with diabetes with better information about medical professional services were more willing to seek help [70]. Accordingly, a positive help-seeking attitude may be related to information on mental illness care resources rather than knowledge of positive mental health. Further studies are required to verify this hypothesis.

The present study has several limitations. First, this study was cross-sectional, and clear causal associations among the five components of MHL could not be determined. Second, the scale is a self-reported measurement, which may be vulnerable to social desirability bias. Finally, the participants were limited to medical and public health students rather than a representative sample of healthcare professionals and students, suggesting that results may not be able to be generalized for health professionals and students in other disciplines. A suggestion for future studies is to investigate the actual help-seeking behaviors of the participants. Moreover, more studies are needed with longitudinal data to better understand the causal associations between help-seeking attitudes and help-seeking behaviors. Such studies may explain why individuals sometimes fail to behave in a way they would like to, even though they have existing and strong intentions [71].

## 5. Conclusions

To conclude, the present study established a theory-based multi-construct of MHL to explain the process of mental health help-seeking among healthcare students. We demonstrated that help-seeking efficacy mediated the relationship between recognition of mental illness and help-seeking attitudes. Healthcare students with better recognition of mental illness improve their help-seeking efficacy, which eventually enhances their willingness to seek help from mental health professionals. These findings have important implications for mental health promotion, which should target help-seeking efficacy in healthcare students. This study adds support to previous literature on mental health education to promote the need to seek help by providing empirical evidence, suggesting that, among Taiwan healthcare students, the effect of recognition of mental illness on mental health help-seeking attitudes is mediated by one’s knowledge about mental health care services. Mental health education would be a crucial step to improve recognition of mental illness, strengthen help-seeking efficacy, and ultimately promote better attitudes toward mental health help-seeking and actual help-seeking behavior.

## Figures and Tables

**Table 1:** Descriptive and inferential statistics of measured variables (*n* = 1294).

Observed Variable	Mean (SD)	Range	Skewness	Kurtosis	Tolerance	VIF
Maintenance of positive mental health	41.52 (5.52)	5–50	−0.52	0.89	0.92	1.08
Recognition of mental illness	16.62 (1.84)	4–20	−0.34	0.77	0.92	1.09
Mental illness stigma attitude	15.37 (3.81)	6–30	0.24	0.24	0.95	1.04
Help-seeking efficacy	11.74 (2.13)	3–15	−0.67	0.78	0.92	1.07
Help-seeking attitude	10.14 (2.42)	3–15	−0.10	−0.14	−	−

Note: VIF = variance inflation factor.

**Table 2:** Direct and indirect effects of 99% confidence intervals for the final model.

	Point Estimates	SE	Z	Bias-Corrected 99% CI	
				Lower	Upper
Direct effect					
R→A	0.08	0.04	2.00	−0.03	0.18
R→S	−0.05	0.04	−1.25	−0.16	0.06
R→HE	0.19 **	0.04	4.75	0.08	0.29
R→M	0.33 **	0.04	8.25	0.22	0.42
S→HA	0.06	0.04	1.50	−0.03	0.16
HE→HA	0.25 **	0.04	6.25	0.15	0.33
M→HA	0.04	0.04	1.00	−0.05	0.14
Indirect effect					
R→S→HA	−0.003	0.004	−0.75	−0.02	0.003
R→HE→HA	0.04 **	0.01	4.00	0.02	0.08
R→M→HA	0.01	0.01	1.00	−0.02	0.05
Total effect					
R→S→HA	0.00	0.00	−1.25	−0.004	0.06
R→HE→HA	0.004	0.002	4.75	−0.001	0.29
R→M→HA	0.001	0.001	8.25	−0.001	0.42

Note: M = maintenance of positive mental health; R = recognition of mental illness; S = mental illness stigma attitude; HE = help-seeking efficacy; HA = help-seeking attitude. SE = standard error; CI = confidence interval. ** *p* < 0.01.

## Data Availability

Due to the nature of this research, participants of this study did not agree for their data to be shared publicly, so supporting data is not available.

## Appendix A

**Table A1:** Direct and indirect effects of 99% confidence intervals for the 26-item model.

	Point Estimates	SE	Z	Bias-Corrected 99% CI	
				Lower	Upper
Direct effect					
R→A	0.08	0.04	2.17	−0.02	0.19
R→S	−0.05	0.04	−1.31	−0.16	0.06
R→HE	0.19 **	0.04	4.75	0.06	0.29
R→M	0.34 **	0.04	8.55	0.22	0.42
S→HA	0.07	0.04	1.94	−0.02	0.16
HE→HA	0.25 **	0.04	7.00	0.16	0.34
M→HA	0.07	0.04	1.89	−0.03	0.16
Indirect effect					
R→S→HA	−0.004	0.004	−1.00	−0.02	0.001
R→HE→HA	0.05 **	0.01	4.00	0.03	0.08
R→M→HA	0.02	0.01	1.83	0.00	0.05
Total effect					
R→S→HA	0.00	0.00	0.00	−0.003	0.00
R→HE→HA	0.004	0.002	2.00	0.00	0.01
R→M→HA	0.002	0.001	2.00	0.00	0.007

Note: M = maintenance of positive mental health; R = recognition of mental illness; S = mental illness stigma attitude; HE = help-seeking efficacy; HA = help-seeking attitude. SE = standard error; CI = confidence interval. ** *p* < 0.01.
